# Magnetic Graphene-Based Sheets for Bacteria Capture and Destruction Using a High-Frequency Magnetic Field

**DOI:** 10.3390/nano10040674

**Published:** 2020-04-03

**Authors:** Andri Hardiansyah, Ming-Chien Yang, Hung-Liang Liao, Yu-Wei Cheng, Fredina Destyorini, Yuyun Irmawati, Chi-Ming Liu, Ming-Chi Yung, Chuan-Chih Hsu, Ting-Yu Liu

**Affiliations:** 1 Research Center for Physics, Indonesian Institute of Sciences, Tangerang Selatan 15314, Indonesia; andri.hardiansyah@lipi.go.id (A.H.); fredinadestyorini@gmail.com (F.D.); irmawatiyuyun@gmail.com (Y.I.); 2 Department of Materials Science and Engineering, National Taiwan University of Science and Technology, Taipei 10607, Taiwan; myang@mail.ntust.edu.tw (M.-C.Y.); firstoneayu@hotmail.com (H.-L.L.); 3 Department of Materials Engineering, Ming Chi University of Technology, New Taipei City 24301, Taiwan; louischengblue@gmail.com (Y.-W.C.); U04187141@mail2.mcut.edu.tw (C.-M.L.); 4 Department of Cardiovascular Surgery, Taiwan Adventist Hospital, and School of Medicine, National Yang Ming University, Taipei 105, Taiwan; mcyung52@hotmail.com; 5 Division of Cardiovascular Surgery, Department of Surgery, Taipei Medical University Hospital, Taipei Heart Institute, Taipei Medical University, Taipei 11031, Taiwan

**Keywords:** magnetic nanoparticles, reduced graphene oxide sheets, high-frequency magnetic field, bacteria capturing

## Abstract

Magnetic reduced graphene oxide (MRGO) sheets were prepared by embedding Fe_3_O_4_ nanoparticles on polyvinylpyrrolidone (PVP) and poly(diallyldimethylammonium chloride) (PDDA)-modified graphene oxide (GO) sheets for bacteria capture and destruction under a high-frequency magnetic field (HFMF). The characteristics of MRGO sheets were evaluated systematically by transmission electron microscopy (TEM), scanning electron microscopy (SEM), zeta potential measurement, X-ray diffraction (XRD), vibrating sample magnetometry (VSM), and X-ray photoelectron spectroscopy (XPS). TEM observation revealed that magnetic nanoparticles (8–10 nm) were dispersed on MRGO sheets. VSM measurements confirmed the superparamagnetic characteristics of the MRGO sheets. Under HFMF exposure, the temperature of MRGO sheets increased from 25 to 42 °C. Furthermore, we investigated the capability of MRGO sheets to capture and destroy bacteria (*Staphylococcus aureus*). The results show that MRGO sheets could capture bacteria and kill them through an HFMF, showing a great potential in magnetic separation and antibacterial application.

## 1. Introduction

Accelerating research on carbon nanostructures led to the discovery of graphene. Structurally, graphene consists of a flat monolayer of carbon atoms tightly packed into a two-dimensional (2D) honeycomb lattice. Graphene is the basic fundamental structure for the derivative graphitic materials of all other dimensionalities including fullerenes, nanotubes, and graphite [1], and is also the thinnest and strongest material. Its charge carriers exhibit giant intrinsic mobility, have zero effective mass, and can travel for micrometers without scattering at room temperature [2]. Currently, graphene has attracted much attention in materials science [3], biotechnology [4], and bio-detection [5]. Significant development has been conducted for the utilization of graphene in bio-applications. Bio-applications of graphene and graphene oxide remain challenging and must be solved with effective collaborations through multiple disciplines, including biotechnology and nanotechnology [6,7].

Among the types of graphene-based materials, graphene oxide (GO)-based materials display a high specific surface area with an abundance of active oxygen-based functional groups including carboxylic, carbonyl, epoxy, or hydroxyl groups around their plane edge and basal plane structure [8,9]. These unique characteristics provide special sites for immobilization and functionalization by various moieties including polymeric materials such as polyethylenimine [10], chitosan [11], poly(lactic acid) [12,13], and inorganic nanostructures including gold [14], TiO_2_ [15], silver nanoparticles [16] and iron oxide nanoparticles [8,14]. Specifically, iron oxide nanoparticles exhibited magnetic characteristics that could be applied to various applications, especially for magnetic fluid hyperthermia [17], magnetic-based bio-separation [18], diagnosis and treatment of infections of bacteria [19], and inductive heating-based magnetic field exposure [20]. Currently, the combination of graphene-based materials and iron oxide nanoparticles have attracted attention due to their potential characteristics as graphene-based materials combined with magnetic nanoparticles [21]. The embedment of magnetite nanoparticles to the graphene sheets could restrict the graphene sheets from the agglomeration and restacking between each layer, promoting the stabilization of graphene layers and the enhancement of the surface area of the graphene sheets.

Currently, graphene-based materials have been extensively studied for their antibacterial activity against various types of bacteria such as *E. coli*, *Colibacillus*, *S. aureus*, or *Candidia albicans* [22,23,24]. Several features of graphene-based materials have been assessed to evaluate their antibacterial activity such as sheet size and concentration, surface area, surface roughness, hydrophilicity, dispersibility, and functionalization [25]. Bo Hu et al. have developed nanohybrid magnetic reduced graphene oxide composed of reduced graphene oxide, Fe_3_O_4_ and a bimetal core-shell of gold/silver (Au-Ag-Au). They proposed the capture, separation, and destruction of *Escherichia coli* by using near-infrared photothermal treatment [26]. Rajni Singh et al. prepared nanocomposites of GO/Fe_3_O_4_ and investigated their characteristics against several pathogenic bacteria and found that GO/Fe_3_O_4_ as an effective bactericidal [8]. Moreover, various antibacterial mechanisms of graphene-based materials have been proposed, such as oxidative stress [27], membrane stress [27], electron transfer [28,29] or wrapping isolation [25]. Omid Akhavan and Elham Ghaderi have investigated the toxicity of graphene oxide nanosheets against *S. aureus* and *E. coli*. They proposed that the edges of the GO nanosheets and the charge transfer between bacteria have a significant effect to destroy the cell membrane of bacteria [30].

In respect to magnetic field exposures, a high-frequency magnetic field (HFMF) has been widely used as a tool to generate a magnetic field for hyperthermia applications [31,32]. Magnetic particles-based hyperthermia has been developed as a method that could potentially be applied to biomedical applications such as tumor therapy [33,34]. Previously, L.-Z. Bai et al. investigated the inductive heating property of GO–Fe_3_O_4_. They found that the temperature of a physiological saline suspension containing GO–Fe_3_O_4_ hybrid could enhanced to 92.8 °C during the AC magnetic field exposure [35]. Szabo et al. have prepared the magnetite/GO nanocomposites through the utilization of strong electrostatic interactions between the magnetite and GO particles [36].

This study will focus on the preparation and characterization of magnetic reduced graphene oxide (MRGO) and evaluate the antipathogenic behavior of MRGO in the exposure of HFMF. Polyvinylpyrrolidone (PVP) and poly(diallyldimethylammonium chloride) (PDDA) were used to modify the surface of GO and subsequently used as a substrate for the immobilization of iron oxide nanoparticles, denoted as MRGO. MRGO sheets were systematically characterized, including their structure, morphology, magnetic properties, and their interaction with bacteria. Specifically, the capturing capability was evaluated against *Staphylococcus aureus* as the model for pathogenic bacteria. The HFMF exposure was conducted to generate the inductive heating effects of MRGO sheets. To the best of our knowledge, there have only been a few reports related to the development of magnetic graphene and investigating its capture of bacteria and hyperthermia treatment simultaneously. Eventually, the combination of capturing and separating bacteria using an external magnetic field and killing them with HFMF-induced effects could potentially be applied in bio-separation and therapy.

## 2. Materials and Methods

### 2.1. Materials

Graphite powder (<20 μm, synthetic), fuming nitric acid (HNO_3_), PVP, and PDDA (Mw < 100,000) were purchased from Sigma Aldrich, St. Louis, MO, USA. Potassium permanganate (KMnO_4_) and sulfuric acid (H_2_SO_4_) were purchased from J.T. Baker Chemical Company, Philipsburg, NJ, USA. Hydrogen peroxide (H_2_O_2_) was purchased from Acros. Hydrochloric acid (HCl) was purchased from Scharlau, Scharlab, Barcelona, Spain. High-purity water purified by a Milli Q Plus water purifier system (Milipore, St. Louis, MO, USA), with a resistivity of 18.3 MΩcm was used in all experiments. All the chemicals were used without further purification. 

### 2.2. Preparation of Magnetic Reduced Graphene Oxide (MRGO)

GO was prepared via modified Hummers method using graphite as the precursor material followed by the oxidation processes [37]. Surface modification of GO was conducted through the direct mixing with PVP and PDDA, simultaneously. Herein, PVP (average molecular weight 10,000 Da) was used to improve the aqueous dispersibility of GO [38]. Briefly, PVP (10 mg/mL) was added into the GO solution (1 mg/mL) as the dispersant. Furthermore, the above PVP-modified GO solution was mixed with 10 mg/mL of PDDA (the stock concentration: 0.2 g/mL) solution and 0.625 M of KCl in the 20 mL of deionized water. The GO-PDDA solution was then stirred for 10 min at 90 °C and then stirred continuously for 12 h at 25 °C. The color change of the GO-PDDA solution confirms the reduction process of GO (brown) transformed to GO-PDDA (black) [37].

**Figure 1:**
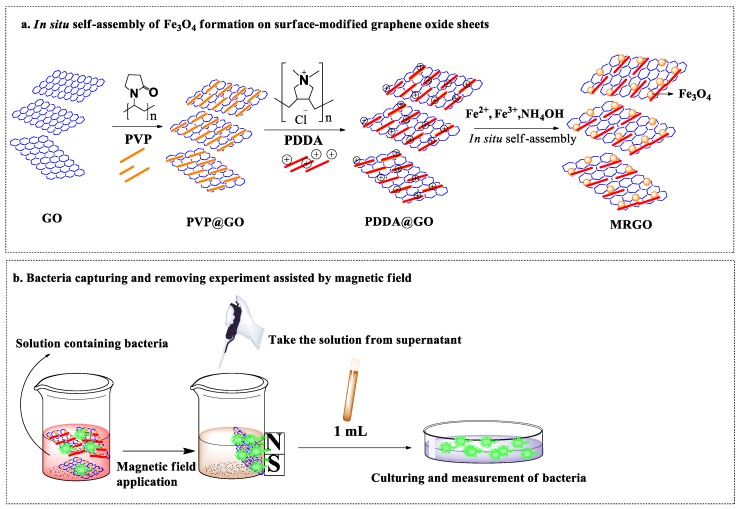
Schematic diagram of (**a**) magnetically reduced graphene oxide (MRGO) preparation and (**b**) bacteria capturing and separation assisted by magnetic field.

FeCl_2_·4H_2_O and FeCl_3_·6H_2_O were used as the initial precursors for the co-precipitation of Fe_3_O_4_ on the GO surface. Briefly, FeCl_2_·4H_2_O (2.5 mmol) and FeCl_3_·6H_2_O (5 mmol), with a ratio of 1:2, were briefly mixed homogeneously at room temperature [39]. The GO solution (1 mg/mL) was mixed with the previous solution and then the temperature was increased to 80 °C. Furthermore, ammonia solution (33%) was added dropwise into the solution to initiate the coprecipitation of Fe_3_O_4_ onto the GO sheets. The final precipitation was denoted as MRGO (Figure 1a).

### 2.3. Characterization

The structure and morphology of MRGO were characterized by TEM (TEM-7650, Hitachi, Chiyoda-ku, Japan) at an acceleration voltage of 100 kV. The binding energy characteristics of GO and MRGO were investigated using X-ray photoelectron spectroscopy (XPS) (ESCALAB 250, Thermo VG Scientific, West Sussex, UK), equipped with Mg Kα at 1253.6 eV at the anode. X-ray diffraction (XRD) was conducted using a D_2_ Phaser BRUKER X-ray powder diffractometer to evaluate the phase of the material by scanning dried powder in the 2θ range of 20–80° with Cu K_α_ (1.5406 Å) radiation. The zeta potential of the graphene-based material before and after polymeric modification was determined through electrophoretic mobility measurements (Horiba Instrument). Vibrating sample magnetometry (VSM) (Lakeshore model 7400) was used to evaluate the magnetization of MRGO as a function of the magnetic field (Oe) at room temperature.

The bacteria capture by MRGO was observed using scanning electron microscopy (SEM) (JSM 5510, JEOL, Tokyo, Japan).

### 2.4. Bacterial Culture Assessment and Bacteria Captured by MRGO

Figure 1b shows the schematic representation of the bacteria capturing experiments by the permanent magnetic field. The bacteria capturing capability of MRGO was evaluated against *S. aureus* (ATCC 43894). Briefly, frozen preserved stock was thawed at room temperature, 0.1 mL was then pipetted and streaked into a quadrant on a nutrient agar plate. Nutrient agar was composed of 6 g agar bacteriological (Agar No. 1) LP0011 powder mixed with 10 g DifcoTM LB (Luria–Bertani) Broth, Miller powder and diluted with 400 mL deionized (DI) water, and cultured at 37 °C for 18–24 h to allow the formation of colonies. Afterward, a single colony was scraped with a loop and swabbed onto a 15°-slant medium (nutrient agar) which was then incubated at 37 °C for 18–24 h. After 18–24 h of culturing, at least 2 mL of NaCl solution (1 wt%) was added and stirred with a vortex mixer for 30 s. After mixing, 1 mL of the solution was added into 9 mL of nutrient broth (5 g Difco™ LB Broth, Miller powder/200 mL DI-water) and mixed with a vortex mixer, then 1 mL of the first nutrient broth solution was mixed into 9 mL of new nutrient broth. This process was repeated four times in a row. An aliquot of 1 mL of nutrient broth containing bacteria (1 × 10^5^ colony forming unit (CFU)/mL) was added to a flask containing 1 mL MRGO, followed by shaking with a rotary shaker at 250 rpm for 20 min. The separation process was conducted by exposing the solution to an external magnetic field for 10 min. After 18–24 h of culturing, the surviving bacteria were counted.

### 2.5. Magnetic Inductive Heating (Hyperthermia) Experiment by HFMF

Magnetic field-induced heat generation of MRGO was generated through HFMF exposure with a frequency of 15 KHz. Briefly, MRGO suspensions were subjected to the center of a copper coil in the HFMF apparatus for 20 min. The temperature increases were recorded every minute using an alcohol thermometer.

## 3. Results and Discussion

### 3.1. Physicochemical Characterizations

**Figure 2:**
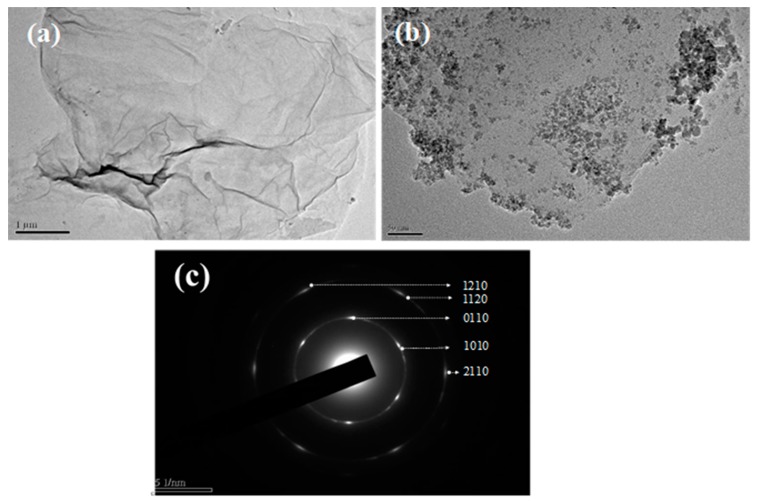
TEM image of (**a**) graphene oxide (GO) (scale bar: 1 mm); (**b**) MRGO (scale bar: 50 nm); (**c**) selected area electron diffraction (SAED) pattern of GO.

Figure 2a-2b shows the TEM images of GO and MRGO, respectively. GO displays the lamellar structures with smooth and wrinkle surface. The diameter of GO is around a few micrometers (2–6 µm) (Figure 2a). Figure 2b shows that the round-like shaped iron oxide (Fe_3_O_4_) nanoparticles with a size of around 8 to 10 nm were embedded onto the surface of GO-based sheets. GO provides the host surface due to its high-surface area for the crystallization of Fe_3_O_4_ nanoparticles, which generated MRGO [21]. Moreover, the oxygen-based functional groups on GO can act as the nucleation sites for the coprecipitation of Fe^2+^/Fe^3+^ [21]. As shown in Figure 2b, the wrinkle structure of GO sheets disappear after the crystallization of Fe_3_O_4_ nanoparticles, which further confirms the reduction process of GO into magnetic reduced graphene oxide (MRGO). This is in accordance with the previous investigation by Bo Hu et al. [26]. Figure 2c indicates the selected area electron diffraction (SAED) pattern of GO, showing the hexagonal structure and the plane (1010) and (1210).

**Figure 3:**
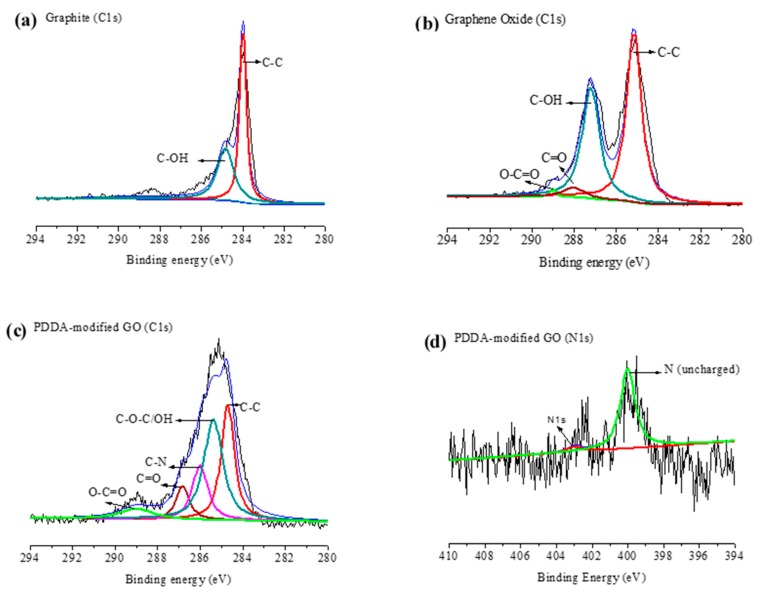
XPS spectra (C-1s) of (**a**) graphite, (**b**) graphene oxide (GO), (**c**) GO-poly(diallyldimethylammonium chloride) (PDDA) (RGO), and (**d**) XPS (N-1s) of GO-PDDA (RGO).

Figure 3 shows the X-ray photoelectron spectra (XPS) of GO and PVP/PDDA-modified GO (reduced graphene oxide, RGO). The C-1s spectra of XPS showed that the graphite contained spectra of C-C at 284.01 eV and C-OH at 284.85 eV, respectively (Figure 3a). Further, the XPS spectra of GO (C-1s) characteristics peaks were found at C-C (285.16 eV), C-O-C/OH (287.17 eV), C=O (287.99 eV), and O-C=O (288.96 eV) (Figure 3b). The relative intensity of the oxygen-based functional group at the C-1s spectra of GO was higher than for graphite. The results confirmed the formation of oxygen-based functional groups, including hydroxyl, carboxylic, and carbonyl groups, on the basal or edge plane of GO as a consequence of the oxidation process on the graphite. Furthermore, the deconvolution C-1s XPS of GO-PDDA (RGO) spectra of C-C, C-O-C/OH, C−N, C=O, and O−C=O were observed at 284.71, 285.40, 286.01, 286.82, and 289.09 eV, respectively (Figure 3c). The presence of N-1s XPS spectra confirmed the immobilization of PDDA (nitrogen-contained polymer) on the surface of GO, as shown in Figure 3d.

**Figure 4:**
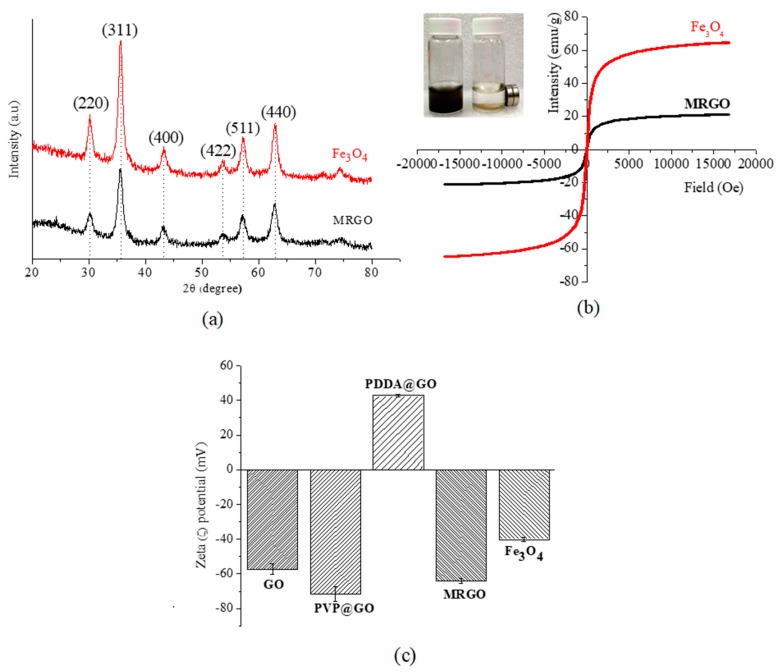
(**a**) XRD pattern and (**b**) VSM curves for Fe_3_O_4_ and MRGO; and (**c**) zeta potential for various as-prepared graphene and Fe_3_O_4_.

Figure 4a–c shows the results of XRD pattern, VSM, and the zeta potential measurement. XRD patterns of Fe_3_O_4_ and MRGO show multiple peaks within the 2θ range of 20–80° (Figure 4a). Six diffraction peaks at 2θ = 30.1° (220), 35.6° (311), 43.3° (400), 53.5° (422), 57.2° (511), and 62.9° (440) were the characteristic peaks of the crystal plane, which was appropriated with the standard diffraction spectrum (JCPDS: 85436). This indicated the formation of magnetite (Fe_3_O_4_) nanoparticles. The XRD pattern of MRGO shows a similar pattern to the pattern of Fe_3_O_4_ nanoparticles, indicating the formation of Fe_3_O_4_ nanoparticles on the graphene surface.

Figure 4b shows the measured saturation magnetization of MRGO and Fe_3_O_4_ nanoparticles by VSM. The magnetization of MRGO was around 20 emu/g, which was strong enough to separate the magnetic graphene from the aqueous solution. The decreasing saturation magnetization value of MRGO in comparison to Fe_3_O_4_ nanoparticles (around 60 emu/g) could be due to the interference of the graphene-based material in the magnetic field exposure. As shown in Figure 4b, the field-dependent magnetization curves are reversible, which confirms that MRGO and Fe_3_O_4_ nanoparticles were superparamagnetic materials with zero coercivity and zero remanence. MRGO could disperse well in the aqueous solution in the absence of magnetic field exposure, which was generated due to the presence of an oxygen-based functional group on the surface of MRGO. Further, MRGO could be collected in the presence of magnetic field exposure (Figure 4b) for the magnetic separation of the microbes.

Figure 4c shows the zeta potential value of each component at pH 7. The zeta potential of Fe_3_O_4_ nanoparticles is −40 mV. This result might be caused by a number of hydroxyl groups (Fe-OH) [40]. GO exhibited negative charged of −60 mV suggesting the successful oxidation process of graphite through the formation of the negatively charged moiety (hydroxyl, epoxy and carboxyl) on the surface of GO. Surface modification of GO using PVP altered the zeta potential value to −70 mV. This may be caused by PVP adsorbing onto the surface of the GO. A positive zeta potential value (+42 mV) of MRGO was achieved after surface modification of PVP/GO by PDDA. PDDA could be tethered onto graphene sheets to develop a net positive charge due to the anchoring of nitrogen-based moiety on PDDA [41]. Eventually, the immobilization of Fe_3_O_4_ onto the PDDA/PVP/GO altered the net zeta potential value to −65 mV, compared with the pristine Fe_3_O_4_ nanoparticles (−40 mV). Thus, highly negatively charged MRGO also proved the high stability of MRGO due to the highly electrostatic repulsion among nanoparticles, which will prevent the aggregation.

### 3.2. Capturing Capability of Bacteria

**Figure 5:**
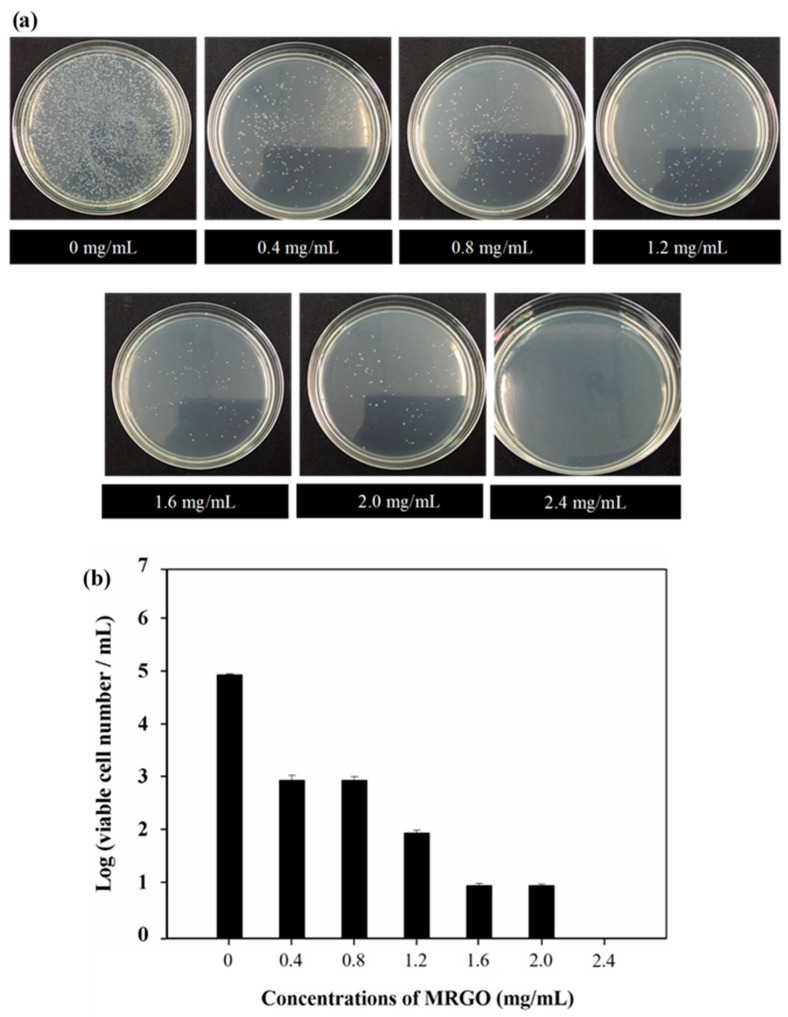
(**a**) Capturing activity toward the supernatant of *S. aureus* (105 CFU/mL) solution with various concentrations of MRGO; (**b**) survival *S. aureus* numbers with various concentrations of MRGO.

**Figure 6:**
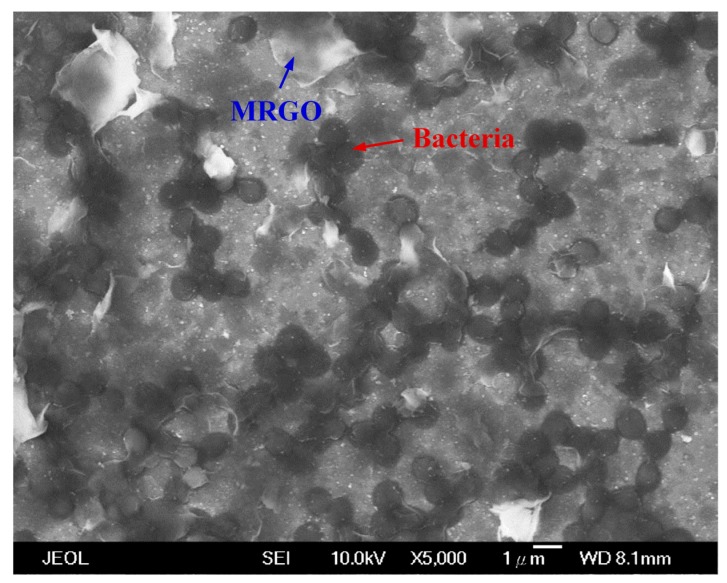
SEM image of MRGO sheets capturing bacteria (*S. aureus*) (scale bar: 1 μm).

Figure 5a shows the optical images of bacteria capturing activity toward the supernatant of *S. aureus* solution with various concentrations of MRGO. The supernatant of bacteria colony decrease indicates that more bacteria was captured. The results show that higher concentrations of MRGO will capture more bacteria. The optimal concentration of MRGO sheets is higher than 2.4 mg/mL, which is enough to collect all of bacteria (10^5^ CFU/mL) (Figure 5b). These results might be due to the higher density of functional groups and surface area of MRGO that could enhance the potential contact and interaction with *S. aureus*, followed by the settlement of bacteria on the surface of graphene-based materials [30]. From the point of view of antibacterial activity, graphene-based materials could damage or break the cell walls and cell membranes of bacteria through the interaction between the sharp edge of graphene-based materials and membranes of bacteria, causing the stress of the bacteria membrane [22,27,42]. This interaction could induce the leakage of RNA and transfer of charge [26]. Previous investigation proposed that the appropriate charge transfer between the bacteria and the more sharpened edges of the graphene-based materials could enhance the antibacterial activity of the graphene-based materials [30]. Another possible mechanism is the isolation of *S. aureus* from the surrounding medium during the incubation with the MRGO; therefore, blocking the consumption of nutrients for proliferation leads to the cells’ death. In Figure 6, SEM images show that the morphology of MRGO sheets and bacteria (*S. aureus*) were captured in the surface of MRGO. A large surface to volume ratio of MRGO provided a space to capture bacteria for the interaction between the MRGO and the cell walls of bacteria. The bacteria were embedded onto the surface of MRGO. On the other hand, magnetic exposure could separate directly the captured bacteria on MRGO in the external magnetic field.

### 3.3. Hyperthermia Experiment and Bacteria Capturing and Removing Activity Assisted by HFMF Exposure

**Figure 7:**
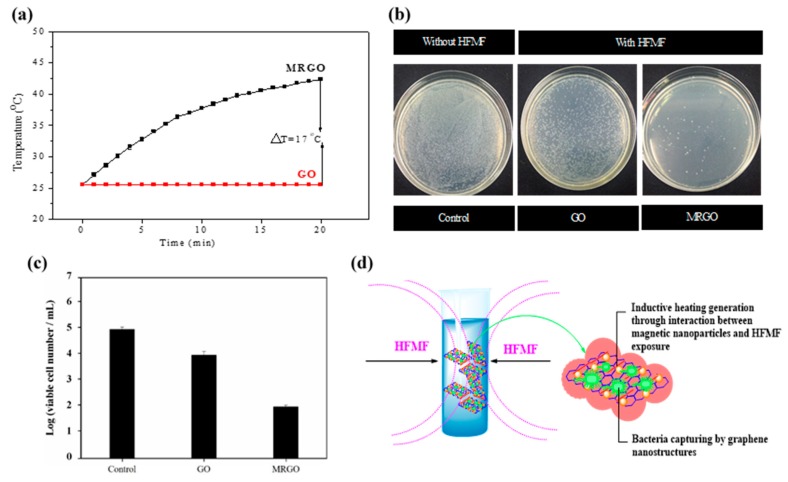
(**a**) Inductive heating temperature of GO and MRGO by high frequency magnetic field (HFMF); (**b**) bacteria activity with and without HFMF exposure; (**c**) survival *S. aureus* numbers of GO and MRGO by HFMF; (**d**) schematic diagrams of bacteria capturing activity and HFMF exposure.

Finally, HFMF was conducted to investigate the magnetic inductive heating of MRGO and to evaluate its capability for killing bacteria. Inductive heating is thermal energy induced from the hysteresis loss of ferrites and is dependent on the type of re-magnetization process in the HFMF [31]. Figure 7a shows the results of the inductive heating ability of GO and MRGO. GO cannot heat up during the exposure to HFMF and the temperature remained around 25 °C (room temperature). Meanwhile, MRGO could increase in temperature up to ~42 °C, after the exposure to HFMF for 20 min. After HFMF was applied in MRGO, it could significantly decrease the colony number of bacteria, in comparison to that in GO (Figure 7b). The bacteria decrease from 10^5^ CFU/mL (control) to ~10^2^ CFU/mL (MRGO) after exposure to HFMF for 20 min, and only 0.1% of bacteria can survive. The calculated capability of MRGO to kill bacteria under HFMF is about 99.9% (Figure 7c). These results confirmed that the temperature increase in the MRGO was closely related to the incorporation of magnetic nanoparticles in the MRGO (Figure 7d). The generation of localized heat could disrupt the surrounding microbes [43].

## 4. Conclusions

We developed an MRGO nanosheet through surface modification of GO using PDDA and coprecipitation of Fe_3_O_4_ nanoparticles onto the graphene sheets for capturing and separating microbes. TEM observation confirmed the formation of MRGO sheets, and Fe_3_O_4_ nanoparticles were able to disperse onto the graphene sheets. MRGO sheets could capture bacteria efficiently. When the content of MRGO reaches 2.4 mg/mL, the bacteria (1 × 10^5^ CFU/mL) can be fully captured by MRGO. Furthermore, the hyperthermia effect, due to the localized heating, could be generated by MRGO through HFMF application and could kill the bacteria. These novel MRGO sheets have the potential to be developed for capturing, separating, and disrupting microbes.
